# Case report: Two cases of juxtapapillary retinal capillary hemangioma treated with intraocular cryotherapy

**DOI:** 10.3389/fmed.2026.1885470

**Published:** 2026-07-06

**Authors:** Weiwei Zhang, Yiliu Yang, Chengda Ren, Xiaoshuang Jiang, Fang Lu

**Affiliations:** Department of Ophthalmology, West China Hospital, Sichuan University, Chengdu, Sichuan, China

**Keywords:** case report, intraocular cryotherapy, juxtapapillary retinal capillary hemangioma, multimodal imaging, pars plana vitrectomy

## Abstract

**Background:**

Juxtapapillary retinal capillary hemangioma (JRCH) is a rare vascular tumor arising from or adjacent to the optic disc and may cause visual impairment due to exudation and vitreoretinal interface complications. Its management remains challenging because of the risk of optic nerve damage and the lack of standardized treatment protocols. Cryotherapy is commonly applied transsclerally for peripheral retinal capillary hemangioma (RCH) but has been rarely used for JRCH. Reports of intraocular cryotherapy are even more limited.

**Case presentation:**

We report two pediatric cases of isolated JRCH with an endophytic growth pattern. The first case involved a 6-year-old girl presenting with JRCH complicated by exudative retinal detachment. She showed a limited response to repeated laser photocoagulation and intravitreal anti-vascular endothelial growth factor (anti-VEGF) therapy, and underwent pars plana vitrectomy (PPV) to relieve vitreoretinal traction. However, peripapillary vascular dilation and exudation persisted. She subsequently underwent intraocular cryotherapy, resulting in tumor regression and reduced exudation, with stable disease maintained during follow-up. The second case involved a 10-year-old girl presenting with JRCH complicated by macular edema and significant vitreoretinal traction. She underwent PPV, epiretinal membrane peeling, and intraocular cryotherapy. The tumor demonstrated a similarly favorable response to intraocular cryotherapy.

**Conclusion:**

Anatomical stabilization was observed during the available follow-up period after treatment including intraocular cryotherapy. These cases provide preliminary clinical experience with intraocular cryotherapy for the management of complex JRCH. Further studies are needed to evaluate its safety, efficacy, and long-term outcomes.

## 1 Introduction

Retinal capillary hemangioma (RCH) is a benign vascular tumor with a reported prevalence ranging from 1 in 40,000 to 60,000 individuals (1). Approximately 50% of cases are associated with von Hippel–Lindau disease (VHL) (1, 2), an autosomal dominant hereditary tumor syndrome, while the remainder occur sporadically. Juxtapapillary retinal capillary hemangioma (JRCH) is a rare subtype of RCH arising from the optic nerve head or its adjacent tissues (3, 4). JRCH predominantly affects pediatric and young adult populations and may lead to painless vision loss due to secondary complications, including exudative retinal detachment, macular edema, and epiretinal membrane formation (5, 6).

In contrast to peripheral tumors, the management of JRCH remains particularly challenging due to its proximity to the optic disc (7). Given this anatomical limitation, laser photocoagulation is effective for small, accessible lesions but has limited utility in the peripapillary region (8–10). For elevated, centrally located, or laser-refractory lesions, photodynamic therapy (PDT) and intravitreal anti-vascular endothelial growth factor (anti-VEGF) therapy have been employed as alternative therapeutic options. However, their efficacy in controlling exudation and preserving visual function remains variable, particularly in lesions adjacent to the optic nerve, where the risk of iatrogenic injury is increased (11–15). In cases complicated by exudative or tractional retinal detachment or vitreous hemorrhage, pars plana vitrectomy (PPV) may be considered. As an invasive procedure, it is generally reserved for more advanced and refractory cases rather than routine management (16–18). Cryotherapy is commonly used for peripheral RCH and has demonstrated efficacy (19–21). It can achieve tumor regression while minimizing damage to adjacent neural structures (22, 23). This technique is typically performed via a transscleral approach. However, due to the difficulty of accessing the peripapillary region through this approach, cryotherapy is rarely applied in cases of JRCH. Therefore, further clinical evaluation is warranted. Here, we report two pediatric cases of JRCH obscuring the optic disc that were managed with treatment including intraocular cryotherapy. Both cases demonstrated tumor regression and anatomical stability during follow-up.

## 2 Case presentation

### 2.1 Case 1

**FIGURE 1:**
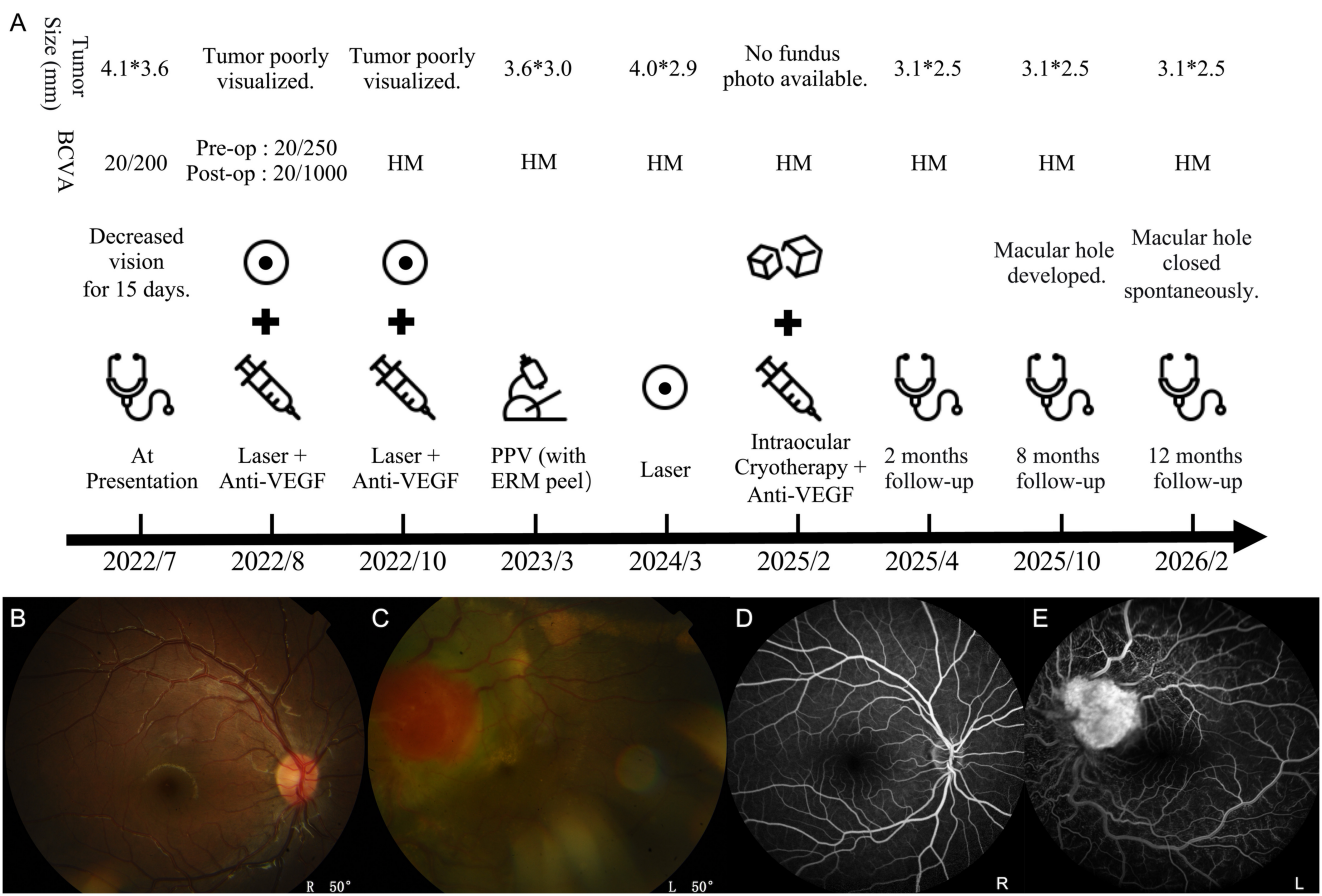
Treatment timeline of patient 1 and multimodal imaging at presentation. **(A)** Treatment timeline. All descriptions refer to the left eye. Tumor size represents the postoperative lesion size at each treatment time point. Pre-op, preoperative; Post-op, postoperative; HM, hand motions; anti-VEGF, anti-vascular endothelial growth factor (anti-VEGF) therapy; PPV, pars plana vitrectomy; ERM, epiretinal membrane. **(B,D)** Right eye showing no abnormalities. **(C)** Fundus photography of the left eye showed a reddish lesion obscuring the optic disc with blurred disc margins and peripapillary vascular proliferation. **(E)** Fluorescein angiography of the left eye demonstrated hyperfluorescence of the optic disc lesion, inferior fluorescein pooling, peripheral vascular leakage, and inferior retinal detachment.

A 6-year-and-5-month-old girl presented to West China Hospital with a 15-day history of painless visual decline in the left eye. She had a history of head trauma more than 1 year ago. The timeline of the clinical course, treatments, and outcomes is shown in Figure 1A.

On initial examination, best-corrected visual acuity (BCVA) was 20/25 in the right eye and 20/200 in the left eye. Intraocular pressure was 14.0 mmHg in the right eye and 10.1 mmHg in the left eye. Anterior segment examination was unremarkable in both eyes, and the right fundus was normal (Figures 1B, D). In the left eye, fundus photography showed a 4.1 × 3.6 mm reddish lesion obscuring the optic disc, with blurred disc margins (Figure 1C). Vitreous opacities, whitish retinal changes and peripapillary vascular proliferation were also observed. Fluorescein angiography (FA) demonstrated marked hyperfluorescence of the optic disc lesion, inferior pooling of fluorescein, peripheral vascular leakage, and inferior retinal detachment (Figure 1E).

To evaluate for underlying VHL disease, a detailed systemic assessment was performed. There was no family history of VHL disease or other hereditary disorders, and brain and orbital magnetic resonance imaging as well as urinary ultrasonography revealed no abnormalities apart from the retinal lesion. Although genetic testing was declined by the family, no evidence of VHL disease or other systemic abnormalities was identified, supporting a diagnosis of isolated JRCH.

**FIGURE 2:**
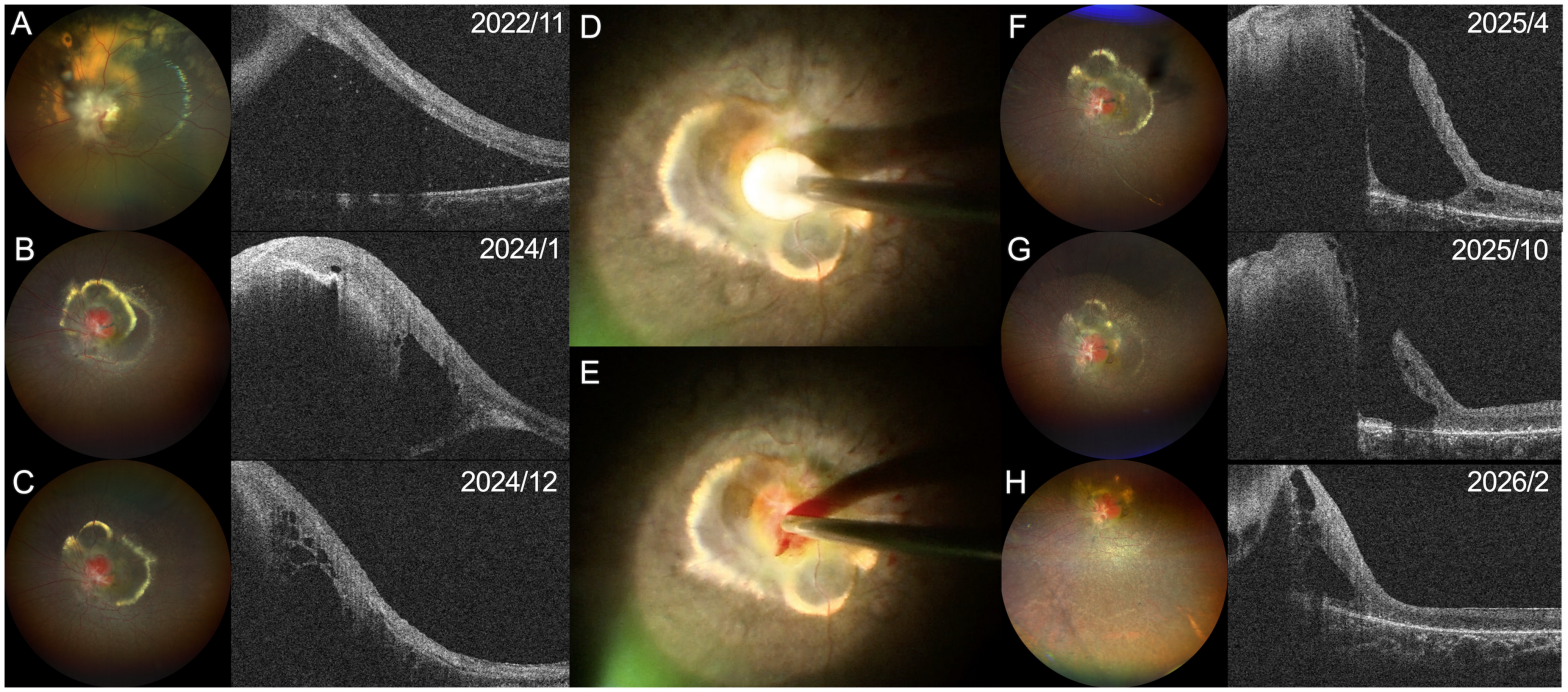
Multimodal imaging of patient 1 after treatment and surgical photographs of cryotherapy. **(A)** After the second session of laser photocoagulation combined with intravitreal conbercept injection, fundus photography showed extensive exudation and prepapillary white fibrous proliferation, and optical coherence tomography revealed total exudative retinal detachment. **(B)** After pars plana vitrectomy, the retinal detachment showed partial resolution. **(C)** After the third session of laser photocoagulation combined with intravitreal conbercept injection, no significant change in exudation was observed, and the peripapillary vessels remained dilated. **(D)** Intraoperative cryotherapy applied to the lesion. **(E)** Minimal intraoperative hemorrhage occurred during the procedure. **(F)** Two months after cryotherapy, tumor regression was observed, with reduced peripapillary vascular tortuosity and dilation, but persistent extensive subretinal fluid remained. **(G)** Eight months after cryotherapy, exudation was reduced, but a macular hole developed. **(H)** One year after cryotherapy, the tumor remained stable with reduced exudation, and the macular hole closed spontaneously.

Before treatment, BCVA in the left eye had declined to 20/250. The patient initially received two sessions of laser photocoagulation combined with intravitreal conbercept injection (0.25 mg; Langmu^®^, Chengdu Kanghong Pharmaceutical Group), an anti-VEGF agent. Laser photocoagulation was performed using a spot size of 150–200 μm, an exposure duration of 0.2–0.3 s, and sufficient power to achieve a grade III gray-white retinal reaction. The treatment achieved temporary tumor control. However, 2 months later, prepapillary white fibrous proliferation and total exudative retinal detachment developed (Figure 2A). BCVA declined to hand motion. A 25-gauge PPV was therefore performed to remove the fibrous proliferation and relieve traction, after which the retinal detachment showed partial resolution. During postoperative follow-up, extensive exudation persisted, and the peripapillary vessels remained dilated (Figure 2B). A third session of laser photocoagulation combined with intravitreal conbercept injection was then administered. However, extensive exudation and dilated peripapillary vessels were still evident at the 9-month follow-up (Figure 2C).

Given the suboptimal response to prior treatment, intraocular cryotherapy combined with intravitreal conbercept injection was performed. Preoperative FA was used to identify the sites of leakage. A 0.89-mm (20-gauge) cryoprobe (Beijing Xinmingren Medical Device Technology Co., Ltd., China) was introduced through a pars plana sclerotomy. The probe tip temperature was set at –80 °C, and cryotherapy was initiated at the most elevated portion of the lesion while avoiding the optic disc whenever possible. Each freeze lasted 8–15 s, and five freeze–thaw cycles were applied until the ice ball completely covered the lesion (Figure 2D), with a total cryotherapy time of 59 s. Minimal intraoperative hemorrhage occurred and required no additional intervention (Figure 2E). The intraocular cryotherapy procedure is further illustrated in Supplementary Video 1. Cryotherapy was followed by an intravitreal conbercept injection. No additional laser photocoagulation was performed during the procedure, and no perfluorocarbon liquid, gas tamponade, or silicone oil tamponade was used.

Two months later, fundus photography showed tumor regression, with the lesion size decreasing to 3.1 × 2.5 mm and reduced peripapillary vascular tortuosity and dilation. However, OCT revealed persistent extensive subretinal fluid (Figure 2F). Eight months later, exudation was reduced, but a macular hole developed (Figure 2G). One year later, the tumor remained stable, and exudation was significantly reduced. BCVA was 20/30 and hand motion in the right and left eyes, respectively. Intraocular pressure was normal in both eyes. Peripapillary vessels showed no abnormality, and spontaneous closure of the macular hole was observed during follow-up (Figure 2H). As no further tumor enlargement or significant exudation was noted, regular follow-up without additional treatment was continued.

### 2.2 Case 2

**FIGURE 3:**
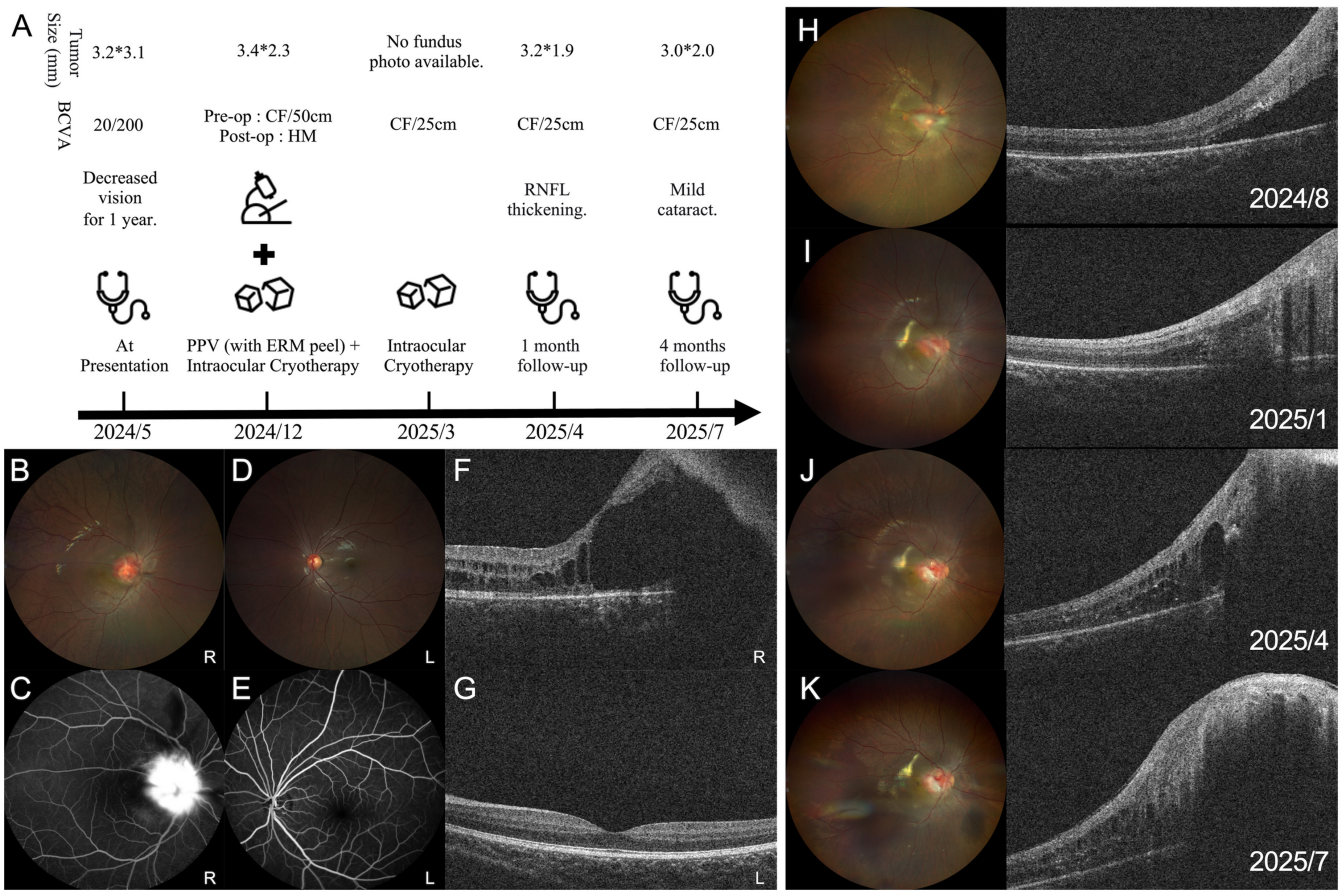
Treatment timeline of patient 2 and multimodal imaging. **(A)** Treatment timeline. All descriptions refer to the right eye. Tumor size represents the postoperative lesion size at each treatment time point. Pre-op, preoperative; Post-op, postoperative; CF, counting fingers; HM, hand motions; PPV, pars plana vitrectomy; ERM, epiretinal membrane. **(B)** Fundus photography of the right eye showed an enlarged reddish optic disc with surface traction and peripapillary vascular proliferation. **(C)** Fluorescein angiography of the right eye demonstrated leakage from the optic disc lesion accompanied by peripapillary hemorrhage. **(F)** Optical coherence tomography of the right eye revealed macular edema and an epiretinal membrane. **(D,E,G)** Left eye showing no abnormalities. **(H)** Two months after the initial presentation, prepapillary fibrous proliferation was observed. **(I)** One month after the first session of cryotherapy, the tumor had slightly regressed, but macular edema persisted. **(J)** One month after the second session of cryotherapy, further tumor regression and reduced exudation were observed. **(K)** Four months after the second cryotherapy, exudation was significantly reduced, and the tumor remained stable.

A 10-year-old girl presented with a 1-year history of painless visual decline in the right eye. She had no history of ocular trauma or systemic disease. The timeline of the clinical course, treatments, and outcomes is shown in Figure 3A.

On initial examination, the BCVA was 20/200 in the right eye and 20/32 in the left eye, with intraocular pressures of 14.0 mmHg and 10.1 mmHg, respectively. Anterior segment examination was unremarkable in both eyes. Fundus photography of the right eye showed a 3.2 × 3.1 mm reddish lesion obscuring the optic disc, with surface traction and peripapillary vascular proliferation (Figure 3B). FA demonstrated leakage from the optic disc lesion accompanied by peripapillary hemorrhage (Figure 3C), while OCT revealed macular edema and an epiretinal membrane (Figure 3F). The left eye was normal (Figures 3D,E,G). In addition, the average peripapillary retinal nerve fiber layer (RNFL) thickness was 68 μm in the right eye and 110 μm in the left eye. There was no family history of VHL disease or other hereditary disorders, and genetic testing for VHL disease was negative. These findings supported a diagnosis of isolated JRCH with macular involvement.

Two months after presentation, prepapillary fibrous proliferation was observed in the right eye (Figure 3H), and BCVA had declined to counting fingers at 50 cm. The patient subsequently underwent 25-gauge PPV with epiretinal membrane peeling and intraocular cryotherapy. Intraocular cryotherapy was performed using the same equipment and settings as described in Case 1. Four freeze–thaw cycles were applied, with individual freeze durations of 1–4 s and a total probe contact time of 12 s. Minor intraoperative hemorrhage occurred but did not require additional intervention. Gross examination of the excised tissue revealed proliferative fibrous tissue with suspected vascular components, indicating a possible vascular origin. One month postoperatively, fundus photography showed slight tumor regression, while OCT demonstrated persistent macular edema (Figure 3I). The average RNFL thickness in the right eye increased to 208 μm, suggesting possible optic disc swelling. To achieve further tumor regression, a second session of intraocular cryotherapy was performed 3 months later. Three freeze–thaw cycles were applied, with individual freeze durations of 12–16 s and a total probe contact time of 40 s. No adjunctive procedures (including laser photocoagulation, perfluorocarbon liquid, gas tamponade, or silicone oil tamponade) were used during either cryotherapy session.

One month after the second session of cryotherapy, the lesion showed further regression with a reduction in size to 3.2 × 1.9 mm and decreased exudation (Figure 3J). The average RNFL thickness in the right eye decreased to 163 μm, indicating improvement of optic disc swelling, while that in the left eye remained stable at 118 μm. Four months after the second cryotherapy, the lesion showed a significant reduction in exudation with the tumor remaining stable in the right eye (Figure 3K), although mild cataract formation was noted. OCT demonstrated marked improvement of the macular edema, with BCVA of counting fingers at 25 cm. The patient continued regular follow-up without additional treatment due to stable tumor size and minimal exudation.

## 3 Discussion and conclusion

JRCH is a rare, benign vascular tumor arising from the optic nerve head or adjacent retina (3). If left untreated, it may lead to visual impairment. According to their growth pattern and retinal layer of origin, JRCHs are classified as exophytic, sessile, or endophytic (24). Exophytic lesions arise from the outer retina and are often associated with subretinal fluid, whereas sessile lesions originate from the middle retinal layers and tend to be relatively flat. In contrast to these two subtypes, both cases in this study were endophytic, arising from the inner retina and extending into the vitreous cavity. The protruding tumors contributed to vitreoretinal traction, epiretinal membrane formation, and exudation, thereby increasing the complexity of surgical management.

Because JRCH is located adjacent to the optic disc and carries a high risk of optic nerve damage, treating JRCH remains challenging. Various treatments for JRCH have been reported, but no clear consensus has been established (9). Representative reports on JRCH treatment are summarized in Table 1.

**TABLE 1:** Representative reports of JRCH: treatments and outcomes.

Author (year)	Type	No. of eyes	Surgical treatment	Anatomical outcome (tumor size)	Functional outcome (best-corrected visual acuity)	Complications	Follow-up (month)
Singh et al. (21)	JRCH (29) RCH (145)	86	Observation (77 tumors) L (43 tumors) TC (39 tumors) NR (15 tumors)	Regression (54/174) Stability (12/174) Progression (108/174)	NR	None	Median 74 (range 0 to 396)
Kreusel et al. (25)	JRCH	1	PPV+ER	Regression	Improved	None	36
Chelala et al. (36)	JRCH	1	Anti-VEGF (ranibizumab)	Stability	Stable	None	6
Papastefanou et al. (11)	JRCH (4) RCH (1)	5	PDT	Regression (3/5) Stability (2/5)	Improved (1/5) Stable (3/5) Worse (1/5)	Epiretinal membrane (1/5) Transient ERD (1/5)	Median 9 (range 4 to 26)
Krivosic et al. (28)	RCH (304)	249	L (297 RCHs) L+TC (7 RCHs)	97% inactivation with L alone vs. 99% with adjunctive TC	NR	NR	Median 138 (range 12 to 504)
Pochop et al. (26)	RCH	9	TTT	Regression (7/9) Recurrence (2/9)	Improved (4/9) Stable (3/9) Worse (2/9)	ERD (2/9)	Mean 126 (range 6 to 216)
Tong et al. (37)	JRCH	1	PDT	Regression	Improved	None	18
Kase et al. (38)	JRCH	1	L	Stability	Stable	None	9
Ucan Gunduz et al. (8)	JRCH	1	L+anti-VEGF (bevacizumab)+PPV	Regression	Stable	None	84
Raval et al. (17)	RCH	16	PPV+ER (7 eyes) PPV+IC/TC (9 eyes)	Regression (14/16) Recurrence (2/16)	Improved (7/16) Stable (4/16) Worse (5/16)	Recurrent RD (8/16) Total cataract (1/16) Phthisis (2/16) Neovascular Glaucoma (1/16)	Mean 29 (range 6 to 79)
Ketkar et al. (27)	JRCH	8	IGC-TTT	Regression (6/8) Stability (2/8)	Improved (1/8) Stable (7/8)	None	Median 11 (range 6 to 29)
Our study	JRCH	2	L+anti-VEGF (conbercept)+PPV+IC (1 eye) PPV+IC (1 eye)	Regression	Improved (1/2) Worse (1/2)	Macular hole (1/2) RNFL thickening (1/2) Mild cataract (1/2)	Mean 8 (range 4 to 12)

JRCH, juxtapapillary retinal capillary hemangioma; RCH, retinal capillary hemangioma; L, laser photocoagulation; TC, transscleral cryotherapy; NR, not reported; PPV, pars plana vitrectomy; ER, endoresection; anti-VEGF, anti-vascular endothelial growth factor therapy; PDT, photodynamic therapy; ERD, exudative retinal detachment; TTT, transpupillary thermotherapy; IC, intraocular cryotherapy; RD, retinal detachment; ICG-TTT, indocyanine green-enhanced transpupillary thermotherapy; RNFL, retinal nerve fiber layer.

Although favorable outcomes have been reported, important limitations remain with existing treatment options. Endoresection following PPV has been reported in isolated cases of JRCH, but its use remains limited because of the risk of injury to adjacent retinal and optic nerve structures (25). Laser photocoagulation is effective for peripheral lesions, but its use near the optic disc is limited by the risk of thermal damage to the retinal nerve fiber layer. In contrast, transpupillary thermotherapy (TTT) uses an 810-nm diode laser with prolonged exposure times and relatively large spot sizes to induce thermotherapy rather than direct photocoagulation. However, TTT may increase exudation and often requires relatively high-power settings. Pochop et al. (26) reported two cases of exudative retinal detachment following TTT, one of which resulted in permanent visual loss despite subsequent vitrectomy. Despite these limitations, indocyanine green-enhanced TTT has recently shown favorable anatomical outcomes in a small series of JRCH cases (27).

Cryotherapy is another established treatment modality, but it is mainly used for peripheral lesions. Via a transscleral approach, cryotherapy induces vascular thrombosis and necrosis through repeated freeze–thaw cycles, achieving effective local tumor control. For peripheral RCH, cryotherapy has been reported to achieve tumor inactivation in approximately 72% of cases, comparable to laser photocoagulation (21). Moreover, Krivosic et al. (28) reported that laser photocoagulation alone achieved tumor inactivation in 97% of RCHs. Among lesions larger than 1 DD, adjunctive cryotherapy improved the inactivation rate from 73% to 94%, resulting in an overall tumor control rate of 99%. These findings suggest that cryotherapy may be useful in managing more challenging or larger lesions. However, complications such as macular scarring and retinal breaks have been reported in 25–27% of cases, mainly in eyes with pre-existing exudation or traction (29, 30). For JRCH, the application of transscleral cryotherapy is limited because the optic disc is difficult to access through an external approach and there are concerns regarding potential optic nerve injury. Although PPV combined with cryotherapy has been reported for complex peripheral RCH and was associated with favorable anatomical outcomes (17), no previous report has described intraocular cryotherapy for JRCH. The present report therefore provides additional experience in this rare clinical setting.

In recent years, PDT and intravitreal anti-VEGF therapy have emerged as alternative treatment options for JRCH, particularly for lesions located near the optic disc. Their effects mainly involve reducing vascular leakage rather than directly inducing tumor regression. Papastefanou et al. (11) reported complications following PDT, including epiretinal membrane formation and exudative retinal detachment. In addition, recent studies have found that PDT may aggravate macular exudation, a condition referred to as PDT-associated acute exudative maculopathy (31). Intravitreal anti-VEGF monotherapy appears to have limited efficacy: Dahr et al. (32) reported reduced fluid leakage without significant tumor shrinkage, while Wong et al. (33) observed continued visual decline with variable tumor response after repeated ranibizumab injections.

Given these limitations, intraocular cryotherapy may represent a useful treatment option in selected cases. In the present report, both pediatric patients presented with relatively large endophytic JRCH completely obscuring the optic disc, making treatment particularly challenging. Within the available follow-up period, both cases achieved anatomical stabilization after treatment including intraocular cryotherapy, with no further tumor enlargement or progressive exudative retinal detachment. However, BCVA improved in one case and declined in the other. This finding is consistent with previous reports that anatomical control of JRCH does not necessarily result in visual improvement, particularly in eyes with poor baseline vision or longstanding macular involvement (11, 27). Several complications were also observed during the treatment course. In Case 1, a macular hole developed and may have been related to vitreoretinal traction, surgical intervention, disease progression, or a combination of these factors (34). In Case 2, transient RNFL thickening suggested possible optic disc edema, which partially improved during follow-up. Cataract progression was also observed and was more likely associated with prior vitrectomy or longstanding exudative retinal detachment than with the cryotherapy procedure itself (35). Minor intraoperative hemorrhage occurred during cryotherapy in both cases but resolved spontaneously without additional intervention.

Traditional transscleral cryotherapy is generally considered an adjunctive treatment and is rarely used in JRCH. In contrast, intraocular cryotherapy enables direct cryoablation of juxtapapillary tumors under intraoperative visualization. Preoperative guidance of FA allows precise targeting of the lesion and vascular occlusion. These features may contribute to the treatment of selected juxtapapillary lesions. However, it should be noted that intraocular cryotherapy is suitable for patients who have already undergone PPV. Otherwise, it may cause iatrogenic vitreous disruption. Additionally, intraoperative hemorrhage may occur as a procedural complication.

Several important limitations should be acknowledged. First, given the small number of cases, these findings cannot be generalized to all patients with JRCH. Second, treatment was heterogeneous and involved multiple interventions in addition to intraocular cryotherapy, and no untreated or natural-history comparison was available. Therefore, the observed anatomical improvements may have been influenced by factors other than cryotherapy, including concomitant treatments, postoperative remodeling, and the natural postoperative course. A causal relationship between intraocular cryotherapy and the observed clinical outcomes cannot be established. Furthermore, follow-up was limited.

In conclusion, this report describes two pediatric cases of endophytic JRCH in which intraocular cryotherapy was used as part of the treatment strategy. During follow-up, anatomical stabilization was observed in both cases, although visual outcomes did not fully correspond to the anatomical findings. Unlike conventional transscleral cryotherapy, intraocular cryotherapy allows direct treatment of the tumor and is typically performed after PPV. Given the challenges of treating lesions involving the optic disc, intraocular cryotherapy may be considered a potential adjunctive option for selected complex cases. Further studies are needed to evaluate its safety, efficacy, and long-term outcomes.

## Data Availability

The original contributions presented in this study are included in this article/Supplementary material, further inquiries can be directed to the corresponding author.

## Supplementary material

Supplementary Video 1 Intraocular cryotherapy for juxtapapillary retinal capillary hemangioma.
