# Supplementary material for: Case report: Two cases of juxtapapillary retinal capillary hemangioma treated with intraocular cryotherapy
Source: Front Med (Lausanne). 2026 Jul 6;13:1885470. doi: 10.3389/fmed.2026.1885470 (PMC13382540; doi:10.3389/fmed.2026.1885470)
Supplement: Supplementary file 1 [file Data_Sheet_1.PDF]

CARE
case report guidelines

CARE Checklist of information to include when writing a case report

(cc) BY-NC-ND

Topic	Item	Checklist item description	Reported on Line
Title	1	The diagnosis or intervention of primary focus followed by the words "case report"	Line 0 (Title)
Key Words	2	2 to 5 key words that identify diagnoses or interventions in this case report, including "case report"	Lines 11-12
Abstract (no references)	3a	Introduction: What is unique about this case and what does it add to the scientific literature?	Lines 15-20
	3b	Main symptoms and/or important clinical findings	Lines 22-23; 28-29
	3c	The main diagnoses, therapeutic interventions, and outcomes	Lines 22-28;30-31
	3d	Conclusion—What is the main "take-away" lesson(s) from this case?	Lines 33-36
Introduction	4	One or two paragraphs summarizing why this case is unique (**may include references**)	Lines 38-60
Patient Information	5a	De-identified patient specific information.	Lines 65; 115
	5b	Primary concerns and symptoms of the patient.	Lines 66;116
	5c	Medical, family, and psycho-social history including relevant genetic information	Lines 76-77;125-127
	5d	Relevant past interventions with outcomes	Lines 81-91
Clinical Findings	6	Describe significant physical examination (PE) and important clinical findings.	Lines 68-75;118-125
Timeline	7	Historical and current information from this episode of care organized as a timeline	Lines 93-104;128-142
Diagnostic Assessment	8a	Diagnostic testing (such as PE, laboratory testing, imaging, surveys).	Lines 76-80;125-127
	8b	Diagnostic challenges (such as access to testing, financial, or cultural)	Lines 79
	8c	Diagnosis (including other diagnoses considered)	Lines 80;127
	8d	Prognosis (such as staging in oncology) where applicable	Lines 105-113;143-150
Therapeutic Intervention	9a	Types of therapeutic intervention (such as pharmacologic, surgical, preventive, self-care)	Lines 81-104;128-142
	9b	Administration of therapeutic intervention (such as dosage, strength, duration)	Lines 82-104;128-142
	9c	Changes in therapeutic intervention (with rationale)	Lines 105-113;143-150
Follow-up and Outcomes	10a	Clinician and patient-assessed outcomes (if available)	Lines 105-113;143-150
	10b	Important follow-up diagnostic and other test results	Lines 105-109;144-149
	10c	Intervention adherence and tolerability (How was this assessed?) Assessed by reviewing medical records and treatment logs.	Lines 93-113;1131-150
	10d	Adverse and unanticipated events	Lines 107; 144-148
Discussion	11a	A scientific discussion of the strengths AND limitations associated with this case report	Lines 229-236
	11b	Discussion of the relevant medical literature **with references**.	Lines 152-206
	11c	The scientific rationale for any conclusions (including assessment of possible causes)	Lines 207-221
	11d	The primary "take-away" lessons of this case report (without references) in a one paragraph conclusion	Lines 222-228
Patient Perspective	12	The patient should share their perspective in one to two paragraphs on the treatment(s) they received	No
Informed Consent	13	Did the patient give informed consent? Please provide if requested	Yes ☑ No ☐
